# Safety implications of remote assessments for suspected COVID-19: qualitative study in UK primary care

**DOI:** 10.1136/bmjqs-2021-013305

**Published:** 2022-03-08

**Authors:** Sietse Wieringa, Ana Luisa Neves, Alexander Rushforth, Emma Ladds, Laiba Husain, Teresa Finlay, Catherine Pope, Trisha Greenhalgh

**Affiliations:** 1 Nuffield Department of Primary Care Health Sciences, University of Oxford, Oxford, UK; 2 Centre for Health Policy, Institute of Global Health Innovation, Imperial College London, London, UK; 3 CWTS, University of Leiden, Leiden, The Netherlands; 4 NIHR CLAHRC Wessex, University of Southampton, Southampton, UK

**Keywords:** general practice, COVID-19, decision support, clinical, patient safety, health services research

## Abstract

**Background:**

The introduction of remote triage and assessment early in the pandemic raised questions about patient safety. We sought to capture patients and clinicians’ experiences of the management of suspected acute COVID-19 and generate wider lessons to inform safer care.

**Setting and sample:**

UK primary healthcare. A subset of relevant data was drawn from five linked in-pandemic qualitative studies. The data set, on a total of 87 participants recruited via social media, patient groups and snowballing, comprised free text excerpts from narrative interviews (10 survivors of acute COVID-19), online focus groups (20 patients and 30 clinicians), contributions to a Delphi panel (12 clinicians) and fieldnotes from an online workshop (15 patients, clinicians and stakeholders).

**Methods:**

Data were uploaded onto NVivo. Coding was initially deductive and informed by WHO and Institute of Medicine frameworks of quality and safety. Further inductive analysis refined our theorisation using a wider range of theories—including those of risk, resilience, crisis management and social justice.

**Results:**

In the early weeks of the pandemic, patient safety was compromised by the driving logic of ‘stay home’ and ‘protect the NHS’, in which both patients and clinicians were encouraged to act in a way that helped reduce pressure on an overloaded system facing a novel pathogen with insufficient staff, tools, processes and systems. Furthermore, patients and clinicians observed a shift to a more transactional approach characterised by overuse of algorithms and decision support tools, limited empathy and lack of holistic assessment.

**Conclusion:**

Lessons from the pandemic suggest three key strategies are needed to prevent avoidable deaths and inequalities in the next crisis: (1) strengthen system resilience (including improved resourcing and staffing; support of new tools and processes; and recognising primary care’s role as the ‘risk sink’ of the healthcare system); (2) develop evidence-based triage and scoring systems; and (3) address social vulnerability.

## Introduction

The US Institute of Medicine’s six dimensions of quality—efficacy, effectiveness, timeliness, patient-centredness, equity and, above all, safety^1^—and the WHO’s International Classification for Patient Safety^2^ were developed in less turbulent times. Their authors assumed, for each illness or condition, the existence of an evidence base, some level of access to basic services, some evidence-based referral criteria and pathways for sick or deteriorating patients, and that staff and systems were not overwhelmed.

Early in the COVID-19 pandemic, these assumptions did not hold. While activity across the National Health Service (NHS) in the UK fell overall, staff had to cope with extensive change and huge physical and psychological risks. The first wave of the pandemic in early 2020 brought a triple challenge: a new disease (whose clinical manifestations were varied, poorly understood and sometimes fatal^3^), rapidly implemented new service models (eg, ‘hot hubs’ in primary care and ‘zoning’ in hospitals for infection control^4 5^) and increased use of technologies due to a shift to assessing patients by phone, video and online symptom checkers.^6–8^ The NHS response to the uncertainties and strains introduced by the pandemic included ‘remote by default’ policies that shifted care away from face-to-face consultation and into virtual domains.

In primary care, English general practices were asked to adopt ‘total triage’ in which people seeking an appointment had to provide information on the reason for consultation by phone or electronically first.^8^ By April 2020, practices had shifted from handling 90% of consultations face to face to undertaking 85% of them remotely.^9 10^ Some practices used a ‘telephone first’ model where every patient spoke to a clinician and a small minority were invited to attend face to face.^8^ In others, various staff including administrators, physician assistants or paramedics did the initial triage. Outside of primary care, the patients used NHS 111 (which offered a free helpline and website for urgent, non-emergency care) to get advice 24 hours/day and book appointments with acute primary care services and accident and emergency (A&E) departments outside of office hours. NHS 111 call handlers, the majority of whom are not clinicians, manage calls using algorithms and the service offered a new COVID-19 National Response Service to assess patients with COVID-19 symptoms. When necessary, the call handlers could refer to a COVID-19 Clinical Assessment Service that provided a remote clinical review, mostly delivered by General Practitioners (GPs).^5^

Little is known about the safety implications of remote assessments for patients with suspected COVID-19. In this paper, we report an analysis of qualitative data selected from five data sets of patients with COVID-19 who were assessed by phone, e-consultation or video during wave 1 (March to June 2020), clinicians who undertook such assessments, and call handlers, support staff and other stakeholders. We asked: what can we learn from their experiences and reflections on the safety effects for individual patients and the wider healthcare system in relation to the shift to remote triage and assessment of suspected COVID-19?

## Methods

### Study design

This study was undertaken on a subset of qualitative data collected for the Remote by Default research study by an interdisciplinary team of clinical and social science researchers from March to July 2020. We combined relevant materials on safety from qualitative interviews with patients, some of whom were also healthcare professionals (data set A), focus groups with patients (data set B), data from an online Delphi study with clinicians (data set C) and focus groups with clinicians and other staff (data set D). These data sets were collected for the purpose of exploring patient, clinician, organisational and system perspectives on the shift to remote assessment. We also included fieldnotes and saved chats from an online workshop combining patient and provider perspectives (data set E). This in-pandemic research identified novel topics such as how to identify people needing urgent escalation of care^11^ and the experience of long COVID.^12^ See table 1 for a complete overview of the aims, sizes and data collection methods of each element of the data set.

**Table 1:** Data sets in the Remote by Default study and subsamples used to explore patient safety issues

Data set label	Sample	Overall aim of this substudy	Recruitment and data collection	Nature of data	Original sample	Subsample analysed in present study on patient safety
A	Narrative interviews with people who had had remote consultations for suspected COVID-19	Exploring experiences on long COVID^12 38^	Patient participants were initially recruited via social media (TG's Twitter feed), through which we made contact with online patient support groups. List managers posted invitations asking specifically for participants from demographic subgroups initially under-represented in our sample (men, older people and ethnically minoritised groups). Further participants, including those not on social media, were recruited by snowballing from primary contacts. Interviews lasted 30–90 min; all were held by telephone or video using Zoom, except for one which was done by email at the participant’s request. Taking a narrative approach,^39^ we interviewed patients asking them to tell their story in their own words and interrupted as little as possible using conversational prompts (such as ‘what happened next?’) to keep the narrative intact and make space for sensitive issues to be raised.	55 interview transcripts	55 patients, of whom 14 were also clinicians	10 individual patient interviews
B	Online focus groups with patients	Exploring experiences on long COVID^12 38^	Patients were recruited by social media and additional snowballing; some were also clinicians. Clinicians were invited to discuss their experiences of assessing patients with suspected COVID-19. Focus groups for patients and clinicians were by Zoom and lasted 90 min. Facilitators used the group process to seek peer reactions to statements and stories and invite similar or contrasting narratives. Video and audio were recorded and contemporaneous fieldnotes taken.	8 focus group transcripts	59 patients, of whom 31 were also clinicians	Focus group comments from approximately 20 patients
C	Delphi study of clinicians including four-round survey	Part of the development of the RECAP severity score 11 33	Clinician participants (including medical consultants, GPs, nurses, paramedics, trainees, emergency specialists) were recruited by social media and additional snowballing. They were invited to provide input to a Delphi exercise via survey (including unlimited space for free text comments); methodological details have been published previously.^11 33^	3 focus group transcripts, free text comments in surveys and on case vignettes	72 (68 GPs, 3 nurses, 1 paramedic)	Free text comments from 30 clinicians
D	Online focus groups with clinicians on assessing COVID-19 remotely with RECAP score	Part of the development of the RECAP severity score 11 33	Focus groups for patients and clinicians were by Zoom and lasted 90 min. Facilitators used the group process to seek peer reactions to statements and stories and invite similar or contrasting narratives. Video and audio were recorded and contemporaneous fieldnotes taken.	3 focus group transcripts	40 (28 GPs, 11 nurses, 1 paramedic)	Focus group comments from approximately 12 clinicians
E	2-hour online workshop for stakeholders	Part of exploration of impact of remote assessment on wider infrastructure^40^	The workshop, the first of a series on the effects of digital system changes on patients and staff in primary care, included clinicians, service users, national policymakers and technology designers; they were invited via email and phone by members of the research team. It was held via Zoom, lasted 120 min and included smaller facilitated breakout groups plus plenaries. We circulated agenda, topics and ground rules about confidentiality and group discussion in advance. Prompt questions for the breakout groups are available from the authors. We took fieldnotes and saved the chats, but did not audiotape the workshop.	Workshop fieldnotes	40 (including clinicians, policymakers, patient reps, technology designers)	Researcher fieldnotes and workshop chat comments from approximately 15 participants
Total					266 participants	87 participants*

*These figures are estimates because it is not possible to be 100% sure which person is speaking in an audio recording of a focus group. We have estimated that approximately 40% of the total sample contributed to the sections analysed for this study.

RECAP, Remote COVID-19 Assessment in Primary Care.

### Selecting data for analysis

Two GP researchers (ALN and SW) of our team read through each data set (table 1) and identified free text material relating to the safety of remote assessments of people with suspected acute COVID-19. ALN and SW discussed these extracts first until they had an agreed selection. Social scientist AR and GP researcher EL, who had also worked on the collection of the data sets, then reviewed, discussed and added to this selection until agreement was reached between researchers on the data for analysis. Relevant data were pasted into interim summary documents including codes to allow us to track back to the wider data set for context.

### Data analysis

After a first familiarisation round, ALN, SW, AR and EL coded the transcripts of talk and fieldnotes with NVivo software. This process started deductively using codes that were informed by the widely used Institute of Medicine’s six dimensions of quality and safety^1^ and the WHO’s International Classification for Patient Safety.^2^ With TG, TF and CP, they then turned to an inductive approach through iteratively discussing the findings in several rounds to elicit further insights and themes using thematic analysis^13^ and drafted a preliminary results section. These results were discussed and refined further by the authors, who studied excerpts from the data sets and synthesised new themes culminating in two broad impacts on patient and healthcare safety. All researchers participated in drafting the final manuscript and selecting illustrative quotes. The paper is being reported according to the Standards for Reporting Qualitative Research reporting guidelines.

### Member checking

A summary of key findings was shared with a sample of 10 participants (five patients and five clinicians) who were randomly selected by a research assistant out of the participants of data sets B, C and D to take account of their comments.

### Management and governance

The study was part of the Remote by Default research programme, funded by the UK Research and Innovation COVID-19 Emergency Fund and a Senior Investigator Award to TG from the Wellcome Trust (which was extended to support pandemic-related work). The study was overseen by an independent advisory group with patient representation and a lay chair who met every 3 months via video link.

## Results

### Overview of data set and findings

The included data sets consisted of 55 individual interviews and transcripts of 14 focus groups (with 171 participants in total) comprising approximately 1450 pages of text. It was evident that the shift towards remote assessment at the start of the pandemic (driven primarily by infection control considerations) was accompanied by a strong pressure on patients to ‘stay home’ and on both staff and patients to ‘protect the NHS’. Facing a novel and deadly disease, they were encouraged to act in a way that helped reduce pressure on an overloaded system with insufficient staff, tools, processes and systems to provide safe primary care, especially for vulnerable patients. Furthermore, patients and clinicians observed a shift from a more or less holistic and adaptive approach in face-to-face consultations to a more transactional (algorithmic, task-oriented) approach when consulting remotely, raising further safety concerns. We consider these themes in more detail below. Participants in member checking broadly agreed with the findings.

1. Pressure to protect the healthcare system (see box 1).

Box 1 **Pressure to protect the healthcare system**

**Overburdened system**

A. *One of my main worries about how the pandemic was handled so far is that they’re so scared that people will be overwhelmed, lots of people didn’t seek help at all because they were told not to or didn’t seek help until too late. I think we, we should be erring on the side of assessing people and assessing people face to face to necessarily, you know, if they might need it and I know we’re trying to find a way that, that makes as little waste as possible but we need to be safe and that’s what’s, something that’s been, that’s been missed throughout all of this and I, I just feel that lots of people have died because they didn’t have any help at all. Sorry, I’m quite upset about it actually.* (Focus group, GP, data set D, RFG2)

**Unprepared for a new condition**

B. *We’re dealing with a disease we haven’t seen before (…) It’s all very well having 40 years’ experience of the health service and knowing what a septic patient presents as but this is quite different it can be, it can catch you out, patients can look well and go off very quickly, they can be unwell when they look well.* (Focus group, GP, data set D, RFG2 R10)

C. *The difficulty is that we have nothing to do to improve things (we are not waiting for antibiotics etc to work), so I am not sure how much people have to deteriorate before they do get admitted.* (Delphi, GP, data set C, ID 11567590814)

D. *It can be quite hard to differentiate between anxiety and an unwell patient over video especially, and paleness and tachycardia makes me concerned regarding shock.* (Delphi, GP, data set C, ID 11576771861)

E. *You’re going to have non-COVID diagnoses which have been misattributed to COVID. So, perhaps someone who has heart failure or someone who has had an MI at home and is then short of breath, or you know, asthma or whatever. I mean literally all of the pathology that existed through COVID-19, but which may trigger a clinician to suspect COVID-19 and therefore encourage the patient to stay at home. So, I think that’s one category of error. But the second category is known complications of COVID-19 not being detected, so things like myocarditis, pulmonary embolism, secondary bacterial pneumonia…* (Interview, patient/clinician, data set A, KT1)

**Inadequate tools and processes**

F. *Because of the lack of ability to score her vital signs, I would be concerned I have ‘underscored’ her.* (Delphi, GP, data set C, ID 11564537389)

G. *In our surgery we have developed a ‘click and collect’ service of a thermometer and a pulse oximeter where patients collect a box with these in from the surgery car park / get dropped of on doorstep in a no contact manner so that we can get them to do the readings at home.* (Delphi, GP, data set D, ID 11565076687)

H. *I tried to get in touch with my GP and he just said: ‘oh you’ve got your own monitor so you’re fine’. And I suppose there was that niggling thing at the back of my mind ‘well I am sure I’m capable of using it but what happens if I’m not?’ It would have been useful maybe to just have a bit of a conversation around it or, you know, I guess ideally I would have loved for a doctor to have put a finger monitor on my finger and just to give me that reassurance that I was doing it right I guess.* (Focus group, patient, data set B, PFG1 R5)

**Access to services**

I. *The doctor at the COVID hub was brilliant. It was a lady and she was really fantastic and to be honest I think just the reassurance that she gave me was probably all I needed. I wasn’t keen to go in by any means, I did not want to go anywhere near the hospital unless it was absolutely 100 per cent necessary but just the fact that she was talking through the symptoms and, ‘Yes okay that’s fine, just keep an eye on it. If this gets worse or that gets worse please call back,’ so I felt like I had the support there if I needed it and, and that kind of reassured me.* (Interview, patient, data set A, IH1)

**Vulnerable groups**

J. *A whole social history is needed – who’s, who’s around, who’s looking after them – because if the people (…) are at risk and there’s nobody there watching over them you have to have a completely different threshold, either having somebody go out and see them face to face or having a paramedic go there for actually getting them into hospital. You’ve gotta do something different (…) depending if they’re on their own or not or, depending who’s with them and whether they’re competent. And that applies to the whole of GPs but I think even more with this because they can deteriorate so quickly.* (Focus group, GP, data set D, RFG2 R2)

### An overburdened, understaffed system

Many clinicians felt frustration and despair at having to deal with or provide what they perceived as unsafe healthcare services. There was widespread concern about the under-resourced and hazardous state of the NHS. Participants believed the driving heuristic for primary care services during wave 1 was reducing referrals to hospital to respond to the reality of limited resources, especially the low availability of testing and the heavy impact of the pandemic on secondary care and emergency services. Some reflected that service efficiency had been achieved at the expense of safety and patient-centredness (box 1, quote A).

### Unprepared for a new condition

Clinical staff noted that the existing guidance and training had not prepared them to deal with this new condition. Remote assessment of patients with suspected COVID-19 was difficult and stressful. Even experienced clinicians found they could no longer rely on their clinical judgement; they described high diagnostic uncertainty, difficulty predicting which patients were likely to deteriorate and clinical impotence (box 1, quotes B and C). They were anxious about misattribution of symptoms to COVID-19 (‘COVID-19 myopia’), and the possibility that they would miss rare complications of COVID-19 and other conditions with similar presentations (eg, anxiety-induced hyperventilation, heart failure or pulmonary embolism) (box 1, quotes D and E).

### Inadequate tools and processes

Clinician participants commented on the difficulty assessing patients remotely and the lack of adequate diagnostic measures, worried that this could result in underestimation of disease severity. For this reason, they felt it was important to provide patients with monitoring tools such as oximeters and thermometers to supplement the information obtainable remotely (box 1, quotes F and G). However, at the time of their acute illness in early 2020, most patient participants did not have an oximeter at home. Even those who did possess an oximeter had not been trained to use one, and contacts with remote clinicians rarely included a conversation about oximetry readings. Because of this, patients felt unsafe and left to their own devices (box 1, quote H). Some patients who were able to provide readings described how clinicians or call handlers dismissed the low oxygen saturation levels they reported.

### Difficulty of service access and navigation

Some patient participants found the NHS 111 telephone triage service and remote GP consultations to be well signposted and easily accessible. Call handlers were ‘helpful’, for example, arranging ambulances or prompting callbacks from a clinician (box 1, quote I). However, most patients described these services as hard to access and inefficient. They reported difficulty navigating e-consultation portals (which required them to complete lengthy online forms) and the NHS 111 telephone service (which transferred them between multiple assessors and asked them to repeat their clinical history several times), with resulting delays in diagnosis and treatment.

### Concern for vulnerable and unsupported groups

Many clinician respondents highlighted that the system for assessing patients with suspected COVID-19 which prevailed in wave 1 had been designed around a somewhat idealised model that cleanly identified ‘low risk’ (safely managed at home) and ‘high risk’ (needing hospital admission) patients. This, they felt, failed to take into account the large numbers of people whose level of risk was moderate or difficult to determine. These included elderly, vulnerable and homebound patients, those with coexisting conditions, those who had difficulties communicating or relied on a relative to give a history and those with low health or digital literacy. Particular concern was expressed for isolated patients who did not realise how unwell they were. Since patients were effectively required to monitor their own deterioration, the presence of a carer could be critical to the disposition decision (box 1, quote J).

2. From holistic to algorithmic and transactional care (see box 2).

Box 2 **From holistic to algorithmic and transactional care**

**Overuse of algorithms**

K. *I decided to get him seen at the hot hub, it didn’t sound like he needed admission but I would, yeah have a look at him in the tent to get some, try and get some more accuracy about the story but I think, I think that with any scoring whatever it relies on, it’s most useful as an aide memoire to address all the different questions particularly when there’s a lot of them a bit like the CHA2DS2VASC [algorithm to calculate stroke risk for patients with atrial fibrillation] […]; it’s also useful if, if it gives you a, a different objective score to your level of clinical concern. You know, if you have a patient with calf pain who’s in a lot of pain and you were worried about a DVT [deep vein thrombosis] and you do the Wells score.* (Focus group, GP, data set D, RFG1 R10)

L. *For all of these kinds of scores the only way they actually get used is if they’re properly integrated with how you carry out your consultations anyway so it’s a kind of user-friendly way. You make it into an EMIS template [an electronic patient record software used in UK general practice] where it auto calculates the score for me, and I’ll use it. I’m probably not going to sit there with it on my desk and work my way through it.* (Focus group, GP, data set D, RFG1 R9)

**Lack of empathy**

M. *I think some of the things, some of the highlights have been when I’d obeyed the Government and gone through 111 and then 999 on the phone and I had a, had a sort of verbal assessment that I wasn’t deemed breathless enough to go to hospital. I was sort of at breaking point because I was so concerned about my breathing.* (Focus group, patient, data set B, PFG1 R7)

N. *But then he didn’t call back and I had to arrange an appointment. And then he was kind of like, ‘Okay, why are you calling?’ And then I had to say the whole story again.* (Interview, patient, data set A, UT1)

O. *[…] so third time lucky I tried to ring 111 and the gentleman I spoke to was, I don’t know if it’s rude or unhelpful, I don’t know what his intentions were, but he told me that until I stopped breathing then there’s nothing they could do about it. And I said ‘You must be joking, do you know what it means to stop breathing? That means I’ve actually died. I can’t, you can’t tell me to stop breathing and then ring you.’ And he said, his words were ‘No, what I meant was you need to be really unwell and really breathless for us. Somebody else [needs] to make that phone call for you.’ And I said ‘Do you realise how many people in this country do not live with, you know, live on their own so if you’re actually waiting for people to, for other people to ring for people, there’ll be lots and lots of people dying at home’. And he was like ‘There’s nothing we can do. You’re not breathless, you need to be breathless for us to come and see you or even for us to escalate your call to the medics’.* (Interview, patient, data set A, NN)

**Value of holistic assessment**

P. *I think for me there are two things, one is asking them why they phoned at that time and in the context of what’s been happening so it’s the trajectory of their, not just their breathlessness but of their, all of their symptoms, but particularly probably their breathlessness.* (Focus group, GP, data set D, RFG3 R10)

Q. *In practice we have noticed that patients are not articulating their breathlessness well - asking them about how their symptoms (both breathlessness and fatigue) are affecting their usual activities has been key - struggling to get out of bed, for example, is often a very significant change.* (Delphi, GP, data set C)

R. *If I see patients face to face I’ll check sat[uration]s or pulse or other observations and record those. But I find mandatory scoring when you look at them and make the decision once you’ve looked at them. After the fact you sort of fit the score to your decision a little bit to almost to reverse justify what you want. It often seems that scoring systems are almost put in our way to remove our autonomy and get us to follow a pathway for everyone which we don’t always agree with. I’m pretty sure I’m not the only GP who thinks like that.* (Focus group, GP, data set D, RFG2 R1)

### Perceived overuse of algorithms and decision support tools; limited enthusiasm for severity scores and symptom checklists

A common complaint of patients was that staff rigidly followed algorithms and scripts that were too narrow and—in their view—sometimes incommensurate with the story they wanted to get across. For example, some participants were told that if they could talk using complete sentences they were not ill enough to need medical attention. Slightly different answers to algorithm questions produced very different dispositions and outcomes, so some participants learnt to ‘game’ NHS 111 or GP calls to access the care they felt they needed (such as summoning an ambulance or managing to speak to a clinician rather than being sent to A&E department).

At the time of our data collection, various guidelines, algorithms and scoring systems were in use to assess severity of suspected COVID-19 and identify patients needing escalation of care. Clinician participants commonly mentioned government guidance on diagnosing COVID-19 (originating from the WHO^14^), which applied a ‘diagnostic triad’ of fever, cough and breathlessness to inform triage questions and prioritise people for swab testing. Widely mentioned resources also included: (1) an early BMJ review article on remote management of COVID-19, which had used hospital-derived data from China to inform a checklist of possible ‘red flags’ and which had argued against using unvalidated scoring systems for breathlessness^6^; (2) rapid guidelines produced by the National Institute for Health and Care Excellence (NICE)^15^ (which drew on the BMJ article); and (3) the UK National Early Warning Score 2 (NEWS2).^16^

Our data illustrated the tension many clinicians felt between using ‘imperfect diagnostic instruments’ and their clinical judgement to assess this new disease (box 2, quote K). Many acknowledged the dangers of over-reliance on instruments they considered too formulaic, not specific to COVID-19 and lacking the depth and nuance of a clinician’s ‘gut feeling’. Some, however, found checklists useful for reminding them of things to look out for, and viewed them as especially helpful for less experienced clinicians (including some trainees). They felt that quantitative scores might provide ‘objective’ data that could then be used (a) as a baseline for further monitoring, (b) for differentiating those who required further assessment, and (c) to support conversations with other healthcare staff, especially when referring to secondary care. They also considered that while red flag signs such as loss of consciousness were usually evident, symptom scoring could be useful for more subtle indicators of severity.

Some respondents believed that a score that aligned well with their clinical assessment and intuition (including ‘eyeballing’ a patient face to face) would increase their diagnostic confidence. Others felt that a score may be most useful when it did *not* support their gut feeling, since it would make them reflect on possible biases. Another concern about severity scores and checklists for COVID-19 was their lack of integration (at the time) into the general practice electronic record, which meant they were poorly aligned with clinical workflows (box 2, quote L).

### Perceived lack of clinical concern and empathy; continuity of care and attention to social support

A minority of respondents reported that clinicians listened patiently, showed empathy, acknowledged uncertainty and provided reassurance or safety netting such as callback or advice about what to do if symptoms worsened (box 2, quote I). Many were told their illness was ‘mild’ and they were being overanxious (one was offered benzodiazepines). Some who went on to experience prolonged symptoms (‘long COVID’) felt that they had been disbelieved and denied adequate care in the acute phase (box 2, quote M).

Some participants commented on being assessed by a doctor who did not know (and, in some cases, could not view) their medical history (box 2, quote N). In such situations, safe triaging and clinical management appeared more challenging, since deterioration could not be monitored against a known clinical or social baseline.

Participants from disadvantaged backgrounds described numerous family and contextual issues that they felt were dismissed by triage staff and clinicians. For example, in one instance, a single parent was told by an NHS 111 call handler to wait and for others to call the services if she could not breathe (box 2, quote O). The impression was that staff were focusing very narrowly on clinical signs and did not engage with wider features that could have a bearing on outcome.

### The importance of traditional history taking and holistic assessment

In quotes P and Q in box 2, clinicians emphasise the value of traditional history taking to draw out a detailed, contextualised narrative of symptoms, speed of deterioration and how the condition is affecting the patient’s ability to function. These quotes also illustrate the emergence of case-based clinical knowledge: both clinicians mention aspects of COVID-19 they had recently learnt to be concerned about, especially disease trajectory and the feeling that severe breathlessness was a helpful symptom if present—but of limited value if absent (an important feature of COVID-19 later described as silent hypoxia^17^). While a few clinicians said they would allow an algorithm or symptom score to over-ride their subjective clinical impression, others described adjusting items in the score to better incorporate more intuitive factors (box 2, quote R).

## Discussion

### Summary of main findings

The first wave of the pandemic in early 2020 brought unprecedented stress to an already overburdened health system. Notwithstanding heroic efforts by individual staff members at all levels,^18–20^ safety concerns arose at both patient level and system level. Clinicians described immense personal stress, uncertainty in assessing the novel and complex presentations of COVID-19 and concern about the lack of measures in place to meet the needs of those with complex needs or limited social support. Their accounts highlight severe understaffing and a system under pressure, with inequities among the vulnerable, isolated and socially disadvantaged. Patients found Remote by Default services difficult to navigate, described feeling ignored and being given impersonal ‘tick box’ care. They perceived an overly rigid adherence to protocols and lack of empathy by some call handlers and clinicians. Health professionals felt ambivalent about these checklists and severity scores.

### Lessons for safer care

Drawing on these findings, we suggest three strategic priorities that we believe will improve patient and system safety as we continue to fight the current pandemic and more generally as we prepare for future crises.

#### Strategic priority 1: strengthen system resilience

##### Prepare for the impactful improbable

Our findings align with a recent paper by Goyal *et al*, who describe how rationing access to healthcare in the early stages of the pandemic had a detrimental influence in the acute phase of COVID-19 in the UK due to the NHS already having insufficient resources, capacity or resilience.^21^ Assessment of the deteriorating patient appears to have been driven by infection control logic (which avoided face-to-face encounters) and demand management (so as not to overwhelm secondary care services) rather than by protocols and pathways designed to provide patient-centred, compassionate care, or optimise outcomes in the individual sick patient.

Supporting safety at organisational and system levels requires acknowledging and building on clinicians and support staff’s ability to function flexibly and effectively under difficult and changing conditions. Our findings resonate with research that showed this was not merely psychologically traumatic but involved moral injury.^22^ Others underscore the importance of adequate staffing^23 24^ to ensure that emergencies are responded to promptly and individual staff do not become overwhelmed and traumatised. At a macro level, a healthcare system must be able ‘to sustain its operations under both expected and unexpected conditions by adjusting its functioning before, during and after events (changes, disturbances or opportunities)’.^25^ Vincent and Amalberti^26^ indicate three strategies for managing risk in an organisation: (a) *avoid* risk in well-controlled environments (eg, using human factor approaches); (b) *manage* risk when these are sufficiently known and understood; and (c) *embrace* risk by working adaptively in unpredictable environments. Safety critical errors are prevented not primarily by tightening procedures but by ensuring that people are aware, mindful, adaptive and creative—and that they are trained and enabled to be so by their organisations.

Our data indicate that in the early weeks of the pandemic, clinicians and patients needed to shift from managing risk towards embracing risk, but they lacked the necessary skills and infrastructure to do so and solutions were overly protocolised. Because the system was close to breaking point, efficiency became over-riding, with a trade-off in terms of safety and patient-centredness. Sustainable strategies should view safety through patients’ eyes and be oriented to achieving patient-centred compassionate care.^26^ This requires patient involvement in adapting services to incorporate their perspectives—a strategy which is now occurring through several online support groups for patients with COVID-19.

##### Support new tools and processes

To deal safely with unexpected events when they occur, new modes of working and investment in diagnostic tools are required. At the time of our data collection, support for home oximetry was not available and patients’ oximetry readings were often dismissed. Major improvements in services have since been made, with all regions of the UK now supporting home oximetry through virtual wards and the COVID Oximetry @home service.^27^ A living systematic review of virtual ward services internationally identified five critical success factors for virtual ward support: resources and training for patients and carers; regular contact from a clinician; measures to compensate for inequalities; clear inclusion criteria, safety netting and escalation pathways; and evaluation and quality improvement.^27 28^

##### Recognise and strengthen primary care’s role as the ‘risk sink’ of the healthcare system

Participants’ general dislike of algorithmic, transactional care underscores the value of traditional history taking using conversational methods (which attend to troubling elements of a story) and holistic assessment of the patient in their family and social context. General practice has been described as the ‘risk sink’ of the NHS,^29^ meaning that under normal circumstances, much clinical risk can be safely contained because patients are known to the practice and vice versa; records are comprehensive, local and up to date; and issues of concern are easily followed up. The pandemic has been described as a ‘fork in the road’ for general practice, potentially heralding major changes in service organisation and delivery that will shift it towards a more transactional model.^30^ Continuity of primary care saves lives generally.^31^ While our study was not designed formally to test this hypothesis specifically in relation to acute COVID-19, our findings strengthen the case for patients being assessed as far as possible by clinicians they know. Our analysis is a reminder that the morality of patient-centred care goes beyond a preoccupation with communication skills and satisfaction measurements. It is a ‘justification in itself, regardless of any measurable relationship with health outcomes’.^32^

#### Strategic priority 2: evidence-based triage and patient-centred scoring systems

Clinical uncertainty and new modes of working require early investment in triage and scoring systems that preserve sensitivity and empathy with patient values and concerns. The checklists and severity scores mentioned by participants had been developed for a variety of uses. The widely cited triad of fever, cough and breathlessness^14^ was (we now know) a very crude diagnostic; applying it as a tool to gauge severity and manage demand at a time when the system was overwhelmed meant that people without these symptoms were de-escalated (eg, advised they were unlikely to have COVID-19 or only had it mildly). The BMJ review^6^ and NICE rapid guidance^15^ were necessarily preliminary (and cautious) in their advice, but clinicians had little else that was specific for COVID-19. NEWS2 was originally developed as a tool for detecting sepsis but recently extended to prehospital care^16^; it had not been validated either for primary care use or for assessment of COVID-19 and should not be used in remote consultations.^33^ Much recent research has been undertaken to improve the sensitivity, specificity and positive and negative predictive values of both diagnostic and severity prediction (prognostic) scores^34^ for safer assessment, but this takes considerable time. The best predictor of whether the patient has COVID-19 is now known to be anosmia, but this is no indicator of severity.^34^ The Remote COVID-19 Assessment in Primary Care score,^33^ developed by our own team, has been integrated with the electronic patient record, and a validation study is ongoing in a sample of 3000 primary care patients.^11^

#### Strategic priority 3: address social vulnerability

Our data on social vulnerability are necessarily partial; our sampling methods and the use of online and telephone data collection methods mean that we have likely not captured the full extent of the interaction between these vulnerabilities and COVID-19. However, we can say that Tudor Hart’s classic inverse care law (which postulates that patients most in need of healthcare are the ones least likely to receive it) also played out at many levels during the pandemic.^35^ Patients with disabilities or caring responsibilities sometimes found it difficult to access care. Accessing new systems and navigating remote consultations and convincing healthcare professionals of need was especially challenging for disadvantaged patient groups including the elderly, those with language barriers and those without digital means. Support for these groups is vital. There is genuine concern that remote delivery might have impaired access to healthcare services and increased inequality.^36^ Further research into the role of translation services and software as well as 24-hour remote support and outreach services to provide these is urgently needed.

### Strengths and limitations of the study

Our study drew on what is, to our knowledge, the largest qualitative research data set on acute COVID-19 collected to date. The range of methods of the primary data sets provided very rich data. We captured the perspectives of a diverse sample of patients and health professionals, with some input from other stakeholders. Experienced qualitative researchers, including clinicians and social scientists, systematically analysed the data drawing on applied theories of healthcare quality and safety. Interpretations were checked with a sample of participants. The study has some limitations, however. Because of pandemic restrictions we were not able to observe consultations directly, or record them. We recruited only a limited number of call handlers and support staff. The study was UK based so should be extrapolated with caution to other countries. It is also possible that our sample may have been skewed towards those patients who had more negative experiences, though the accounts resonate with other accounts of the NHS crisis and mortality figures that prevailed at the time. For example, an independent audit of calls for suspected COVID-19 to the NHS 111 telephone advice line in England, initiated after staff themselves raised concerns, found that 60% of calls did not meet the criteria to be classed as ‘safe’.^37^

In conclusion, it is reassuring that many threats to patient and system safety related to remote assessment of patients with suspected COVID-19 identified in this study have already begun to be addressed. The question of poor system resilience, however, requires wider recognition and urgent action as the NHS contemplates future uncertainties and risks either from the current pandemic or new threats.

## Data Availability

Data are available upon reasonable request. Data are available upon reasonable request from the corresponding author.
